# Dermoscopic Features of Pigmented Bowen's Disease in a Japanese Female Mimicking Malignant Melanoma

**DOI:** 10.1155/2010/543091

**Published:** 2010-08-02

**Authors:** Takayuki Inoue, Ken Kobayashi, Mizuki Sawada, Sumiko Ishizaki, Haruo Ito, Mariko Fujibayashi, Masaru Tanaka

**Affiliations:** ^1^Department of Dermatology, Tokyo Women's Medical University Medical Center East, 2-1-10, Nishi-Ogu, Arakawa-ku,Tokyo 116-8567, Japan; ^2^Department of Pathology, Tokyo Women's Medical University Medical Center East, 2-1-10, Nishi-Ogu, Arakawa-ku, Tokyo 116-8567, Japan

## Abstract

Various structures have been reported for dermoscopic features of pigmented Bowen's disease (BD), which could be a mimic of various pigmented skin lesions. A 79-year-old Japanese woman presented with a 3-year history of brown-black macule on her right upper arm without symptom. Dermoscopic examination demonstrated irregular flossy streaks, irregular brown dots/globules, blue-whitish regression structures, and overlaying whitish scaly areas. We suspected pigmented skin lesions including seborrheic keratosis, pigmented eccrine poroma, and malignant melanoma and excised completely with a 5 mm margin. Histopathological features were consistent with a diagnosis of pigmented BD. Although similar dermoscopic features might be revealed in pigmented skin lesions and it may occasionally be difficult to distinguish between pigmented BD and other pigmented skin lesions, dermoscopy would be useful in speculating pathologic features of pigmented BD.

## 1. Introduction

Dermoscopy not only improves the diagnostic accuracy in pigmented skin lesions, but it is also useful in the evaluation of non-pigmented skin lesions, as it allows the recognition of vascular structures that are not visible to the naked eye. Bowen's disease (BD) or squamous cell carcinoma in situ is usually nonpigmented but may also occasionally be pigmented. Dermoscopy of pigmented BD often shows nonspecific dark pigmentation, which is a mimic of various skin conditions including seborrheic keratosis, pigmented eccrine poroma, and malignant melanoma. We encountered one Japanese patient who had a diagnosis of pigmented BD and evaluated dermoscopic features using nonpolarized, contact-type dermoscopic instrument with echo gel, Derma9500 (DMI, Inc., Yokohama, Japan), in combination with PowerShot 630A (Canon, Tokyo, Japan).

## 2. Case Report

**Figure 1:**
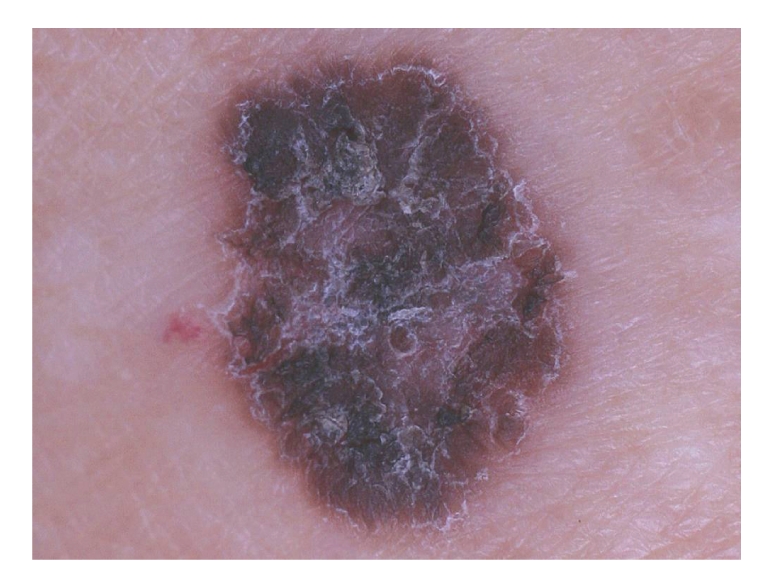
A solitary oval shaped brown-black macule of 8.7 × 11.1 mm with whitish scaly surface was seen on the right upper arm.

A 79-year-old Japanese woman presented with a 3-year history of gradually enlarging macule on her right upper arm. The lesion had no symptom. The patient had no familial and personal history of cutaneous malignancy. Her past medical history included uterine tumor for which she had undergone a hysterectomy 40 years ago. On physical examination, solitary oval shaped brown-black macule of 8.7 × 11.1 mm with whitish scales was detected on the right upper arm (Figure 1). No other skin lesion was noticed, except for vitiligo vulgaris on the right arm. 

**Figure 2:**
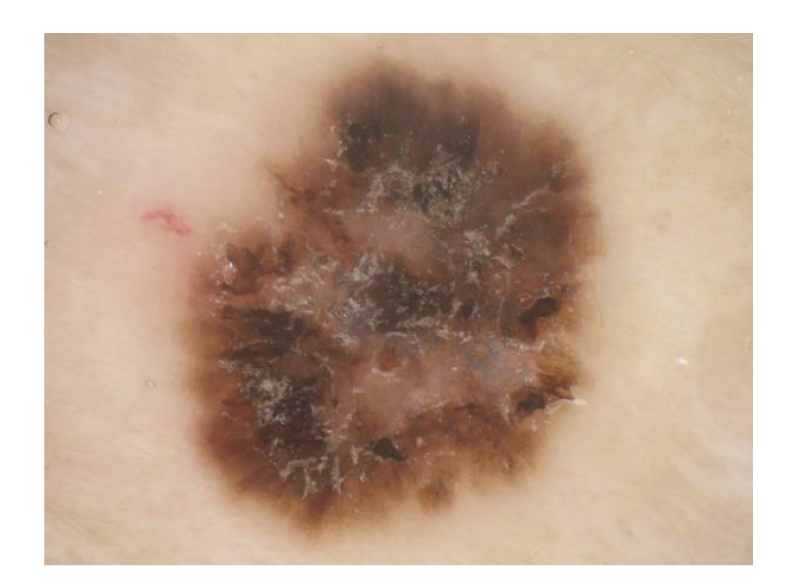
Dermoscopic examination demonstrated irregular flossy streaks, irregular brown dots/globules, and blue-whitish regression structures with overlying whitish scaly areas.

Dermoscopic examination demonstrated light to dark irregular pigmentation with milky red areas containing irregular blue-white structures suggesting partial regression and overlaying many linear and small reflecting whitish scaly areas (Figure 2). Some parts also had irregular brown dots/globules and peripheral bleary irregular streaks. However, no obvious glomerular and/or dotted vessels can be detected. Dermoscopic examination was nonspecific darkly skin lesion. Therefore, we suspected a kind of pigmented skin lesion such as seborrheic keratosis, pigmented eccrine poroma, or malignant melanoma and excised completely with a 5 mm margin. 

**Figure 3:**
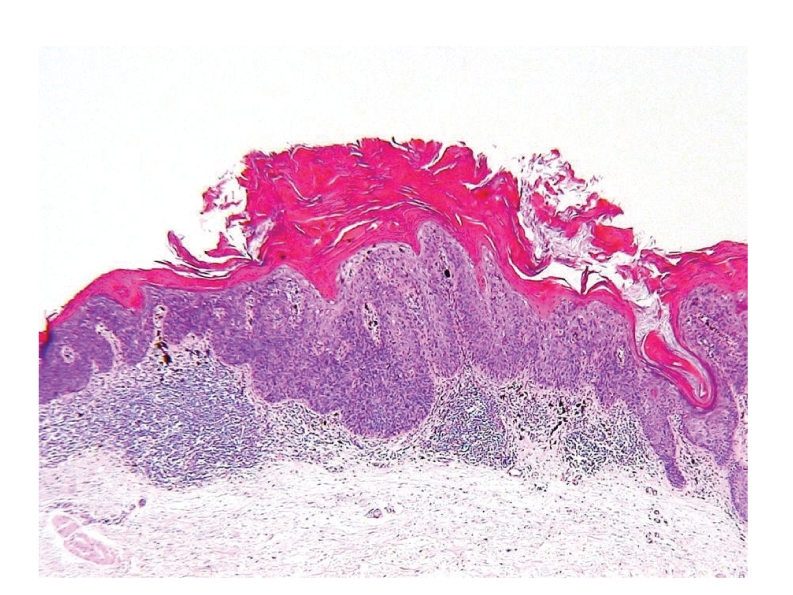
Hematoxylin and eosin staining revealed marked acanthosis with hyperkeratosis and irregular melanin pigment distribution throughout the epidermis to papillary dermis. The papillary dermis showed a dense infiltrate of lymphocytes and numerous scattered melanophages.

Histopathological examination of hematoxylin and eosin-stained specimen (Figure 3) revealed that the epidermis showed marked acanthosis with elongation and thickening of the rete ridges with hyperkeratosis, and marked atypia of keratinocytes. Some keratinocytes had large, hyperchromatic nuclei and contained coarse keratohyaline granules. Atypical mitoses were also observed. Irregular melanin pigment distribution throughout the epidermis was seen, and basal melanosis was also irregularly demonstrated. There was no evidence of dermal invasion. The papillary dermis showed a dense infiltrate of lymphocytes and numerous scattered melanophages. The histopathologic features were consistent with a diagnosis of pigmented BD. 

Pigmented BD is rare and clinically mimics other darkly pigmented lesions such as seborrheic keratosis, pigmented eccrine poroma, and malignant melanoma [1, 4]. Dermoscopic features of Pigmented BD are composed of three main structures (pigmented structures, surface structures, and vascular structures) according to previous papers (Table 1) [2, 3]. Frequent dermoscopic features of pigmented BD included small brown globules regularly packed in a patchy distribution (85%; 11/13), gray to brown homogeneous pigmentation (62%; 8/13), glomerular vessels (77%; 10/13), and scaly surface (69%; 9/13) [1–3]. Cameron et al. [5] reported that linear arrangement of brown and/or gray dots and/or coiled vessels is a specific clue to pigmented Bowen's disease. However, the present case did not demonstrate glomerular and/or coiled vessels, nor linear arrangement of dots. We estimated that the vessel structures and linear arrangement of dots were hidden because our case had marked melanin deposition in the epidermis to papillary dermis.

 In our case of pigmented BD, additional dermoscopic features, namely, peripheral irregular flossy streaks, irregular brown dots/globules, and regression structures were seen. Histopathologically, irregular flossy streaks may correspond to the presence of melanin pigment in the epidermis [6], and irregular brown dots/globules may be related to the presence of melanin pigment in the horny layer to epidermis. Regression structures composed of irregular whitish and bluish areas on the dermoscopy may correspond histopathologically to the aggregated melanophages in the papillary dermis. Whitish scaly areas, namely, many small pieces of whitish reflecting structures may conform histopathologically to parakeratotic horny layers.

Dermoscopic features of pigmented BD are variable and often nonspecific due to dark pigmentations. Therefore, the findings often mimic other pigmented skin lesions including seborrheic keratosis, pigmented eccrine poroma and malignant melanoma (Table 2). In our case, similar dermoscopic features were revealed such as irregular pigmentation, brown dots/globules, and blue-whitish structures, and it may occasionally be difficult to distinguish between pigmented BD and other pigmented skin lesions.

 In conclusion, dermoscopy is indispensable as a helpful tool for increasing diagnostic accuracy of clinical diagnosis of pigmented skin lesions. In pigmented BD, irregular flossy streaks should be added as a criterion especially in dark skin individuals, in addition to brown dots/globules, gray to brown homogeneous pigmentation, glomerular vessels, and/or scaly surface. However, further study is needed to evaluate the specificity and sensitivity of dermoscopic features of pigmented BD for a differential diagnosis from other pigmented skin lesions.

## Figures and Tables

**Table 1:** Summary of previously reported dermoscopic features for pigmented BD [1–3].

(A) Pigmented structures		
(1) Brown globules	85%	(11/13)
(2) Homogeneous pigmentation	62%	(8/13)
(3) Pigment network	15%	(2/13)
(4) Streaks	8%	(1/13)
(5) Regression structures	8%	(1/13)

(B) Surface structures		

(1) Scaly surface	69%	(9/13)
(2) Verrucous surface	8%	(1/13)

(C) Vascular structures		

(1) Glomerular vessels	77%	(10/13)

**Table 2:** Differential diagnoses of pigmented BD on dermoscopy.

	Vessels	Streaks	Dots/globules	Pigmentation
Pigmented BD	glomerular	irregular flossy	irregular brown	diffuse brown
Seborrheic keratosis	comma, hairpin	—	—	opaque brown
Malignant melanoma	polymorphous	irregular	irregular black	irregular black
Pigmented eccrine poroma	hairpin with white halo	—	—	irregular black

## References

[1] Krishnan R, Lewis A, Orengo IF, Rosen T (2001). Pigmented Bowen’s disease (squamous cell carcinoma in situ): a mimic of malignant melanoma. *Dermatologic Surgery*.

[2] Hernandes-Gil J (2008). Clinical and dermoscopic features of pigmented Bowen disease. *Actas Dermosifiliogr*.

[3] Zalaudek I, Argenziano G, Leinweber B (2004). Dermoscopy of Bowen’s disease. *British Journal of Dermatology*.

[4] Stante M, De Giorgi V, Massi D, Chiarugi A, Carli P (2004). Pigmented Bowen's disease mimicking cutaneous melanoma: clinical and dermoscopic aspects. *Dermatologic Surgery*.

[5] Cameron A, Rosendahl C, Tschandl P, Riedl E, Kittler H (2010). Dermatoscopy of pigmented Bowen's disease. *Journal of the American Academy of Dermatology*.

[6] Hayashi Y (2009). Dermoscopy of pigmented Bowen’s disease mimicking early superficial spreading melanoma. *Case Reports in Dermatology*.

